# How Policy Mix Choices Affect the COVID-19 Pandemic Response Outcomes in Chinese Cities: An Empirical Analysis

**DOI:** 10.3390/ijerph19138094

**Published:** 2022-07-01

**Authors:** Chunyu Shi, Tao Xu, Zhihang Ying, Huan Li

**Affiliations:** 1 Department of Public Administration, School of Public Management, Zhejiang Gongshang University, Hangzhou 310018, China; shichunyu@zjgsu.edu.cn; 2 Department of Land Resources Management, School of Public Management, Zhejiang Gongshang University, Hangzhou 310018, China; 1920040103@pop.zjgsu.edu.cn (T.X.); 2020100135@pop.zjgsu.edu.cn (Z.Y.)

**Keywords:** policy mix, policy design, COVID-19, pandemic management, policy outcomes, compound crisis

## Abstract

Since January 2020, the COVID-19 pandemic has caused millions of deaths and has posed a major public health threat worldwide. Such a massive and complex crisis requires quick and comprehensive policy responses. We developed an empirical dataset of policy mixes that included 4915 policies across 36 Chinese cities and investigated the relationships between the policy design choices and the COVID-19 pandemic response outcomes of a city. Using topic modeling and ordinary least squares regression analysis, we found considerable variation among cities in the compositions and design features of their policy mixes. Our analysis revealed that restriction measures did not significantly influence limiting the spread of the pandemic, but they were negatively correlated with the economic growth rate. By contrast, health protection measures greatly contributed to controlling viral spread. Intensive socioeconomic support reduced the occurrence of secondary disasters. The most effective policy strategy to deal with the COVID-19 pandemic appears to be a comprehensive policy design with a mix of restrictions, health protection measures, and socioeconomic support policies accompanied by a timely lockdown. Our empirical findings can help to improve pandemic policy design and contribute to generating broader lessons for how local governments should deal with similar crises in the future.

## 1. Introduction

The coronavirus disease 2019 (COVID-19) pandemic has caused millions of deaths since January 2020 and is a major public health threat worldwide. Such a massive and complex crisis has exposed differences in the capacities of governments around the world to integrate and coordinate different policy tools to manage the pandemic and to deal with its consequences [1,2]. The pandemic offers a natural experiment wherein the crisis that governments faced was broadly the same, but the policy solutions that they enacted were different [2], creating a unique opportunity for understanding associations between policy responses and their consequences; thus, lessons can be learned to improve similar public health risk response capacity [3].

Previous studies have mostly focused on the comparison of state-level pandemic policy responses among countries [2]. They have characterized the “standard” portfolio of national pandemic responses and have discussed the similarities and differences between the various policy tools deployed [2,4]. Scholars have examined these COVID-19 policy responses from institutional and political perspectives [5]. Some studies have included a large-N sample, cataloging the policy tools adopted by governments around the world, and have analyzed the sequence, intensity, and balance of different policy mixes [6,7,8]. However, these studies did not assess how the policy choices impacted the pandemic management outcomes. Other studies have been more comprehensive. They have assessed governments’ responses in detailed case studies [9,10,11], investigating not only how national responses differed but also how these choices were shaped by specific political systems and administrative traditions such as the nature of national leadership; the organization of central and local governments; and the relationships between decision-makers, elites, epistemic communities, and others [12,13,14].

These studies have provided a general understanding of the variety of policy choices among countries and have made important contributions to enriching our knowledge of the various COVID-19 pandemic responses. However, these studies have mostly focused on national policies, and to date, we lack an appreciation of how local governments managed to coordinate their policy tools to deal with the crisis; the consequences of their different policy choices also remain unclear [2,15,16]. The COVID-19 pandemic has been most acutely felt at the local level, especially in cities, where the response policies have been impactful and have significantly affected the COVID-19 crisis management outcomes [17]. For instance, within a country with standardized national response policy guidelines, why did some locations experience recurrent outbreaks while others did not? How did local authorities differ in the attention they gave to other issues that occurred during the pandemic? To clarify what happened in local areas and understand why there were differences in outcomes, we need to closely study local-level policies [18] to supplement and enrich existing findings based on investigations of national-level responses to COVID-19.

This study selected 36 Chinese cities to empirically assess and gain insights into local-level responses to the COVID-19 pandemic. Through the conceptual lens of a policy mix [19,20,21,22], we analyzed 4915 policies that were adopted by these cities between 23 January 2020 (after the lockdown of Wuhan) and 1 April 2022. Following Nauwelaers et al. [19], we defined “policy mix” as a combination of policy tools that interact to produce a specific policy response to resolve complex problems. We investigated how different municipal governments coordinated and integrated their policy tools in mixes to deal with the pandemic, explored links between policy design choices and the cities’ COVID-19 pandemic response outcomes, and discussed how policy mix design can affect the crisis management outcomes in a city.

## 2. Materials and Methods

### 2.1. Data Collection and Sample

Data on COVID-19 response policies were compiled by collecting information from each municipal government’s official website, which provides information on the municipality’s pandemic response measures. The number of outbreaks and that of high-risk areas were retrieved from each city’s Center for Disease Control (CDC) website. As there are no specific official criteria for COVID-19 outbreaks in China, when the virus spreads across administrative jurisdictions, specifically city districts, and the municipal government adopts a full-scale lockdown, we take that as the occurrence of an outbreak. High-risk areas are defined as counties or urban districts having more than 50 cumulative cases or clustered infections in the past 14 days (see the Guidelines on COVID-19 Prevention and Control issued by the Joint Prevention and Control Mechanism of the State Council, 17 February 2020).

The total number of secondary accidents caused by the pandemic control measures (including food shortages, accidental deaths due to refusal of access to medical treatment, the killings of dogs and cats, violent confrontations between officials and citizens, and other immediate damage) in each city were collected from a website where people can see daily trends in micro-blog topics in China (www.weibotop.cn/2.0/#, accessed on 2 April 2022). This is a palliative due to the lack of official statistics on this issue. Thus, these data are approximate and reflect the overall severity rather than the true total numbers of secondary accidents.

Information on the cities’ 2-year average economic growth rates, gross domestic product (GDP), total population, and universities were collected from the National Bureau of Statistics of China website (https://data.stats.gov.cn, accessed on 21 March 2022). The cities’ digital city rankings were obtained from a China Center for Information Industry Development (CCID) report published in June 2021 [23]. 

### 2.2. City Selection

**Figure 1:**
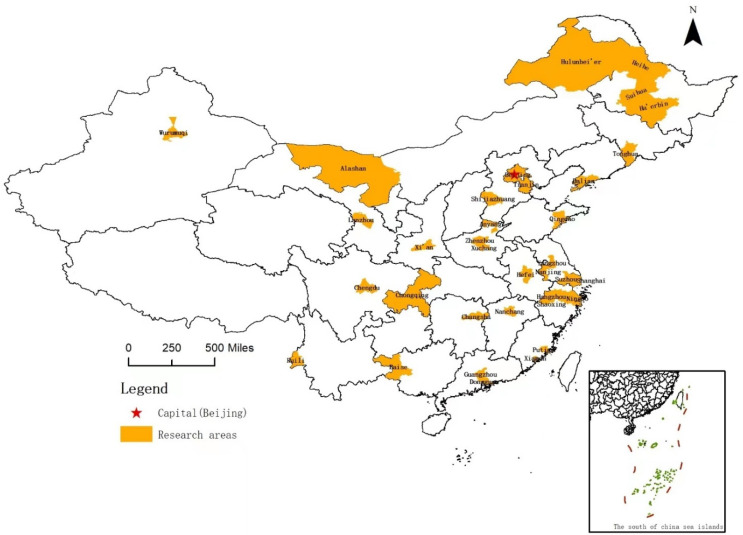
The locations of the 36 cities investigated in this study.

The 36 cities (Figure 1) included in this study were selected mainly for the following reasons:

1. All 36 cities are municipal-level cities (divided into districts). According to the current Law on the Prevention and Treatment of Infectious Diseases, the municipal government is the agency responsible for responding to infectious diseases within its jurisdiction. It is the key decision-making body for pandemic management. The central or provincial governments only intervene when the municipal government is incapable of controlling the crisis. In that case, the higher levels of government primarily act as coordinators and supervisors but do not replace the municipal government in directly managing the crisis.
2. These 36 cities represent different geographical locations, population sizes, economic and technological development levels, and administrative and cultural traditions; they are located in eastern, central, and western China. Among them, there are 4 municipalities directly under the central government; another 14 are provincial capitals, and the other 18 are important transportation hubs, with active international economic and trade exchange. These hubs are close to mega-cities or are border cities. Although the cities differ considerably, they all face high pressure to prevent and control the COVID-19 pandemic.
3. The cities have relatively independent discretion to deal with the pandemic. Thus, our sample cities are comparable yet representative for examining the relationships between the different policy strategies that were chosen and the pandemic management outcomes of those choices.

### 2.3. Analysis Methods

This study adopted a comparative methodology to shed light on the link between policy design and the cities’ pandemic response outcomes. First, we used structural topic modeling to identify the topics around which the COVID-19 policy responses were clustered. Topic modeling is a machine learning technique for discovering latent common topics in text documents [24,25]. The topics resulting from this analysis were interpreted as types of policy tools adopted by municipal governments to deal with the COVID-19 pandemic. We applied the topic modeling in the following steps: (1) we collected documents related to municipal pandemic prevention and control policies and compiled them into a policy dataset; (2) we processed the word segmentation, then removed stop words in the Chinese language as well as frequently occurring words in this dataset (for example, “announce”, “COVID-19”, “policy”); and (3) we employed collocations to create a model for phrase extraction and for optimizing the word segmentation results. (4) After preprocessing the text, we selected the number of topics (*k* = 28) based on the assessment of models with 4–30 topics. Running the topic model, we obtained the prevalence of each policy tool, and the sum of the prevalence of the overall types of policy is always 1. The topic prevalence refers to the proportion of each topic in the corpus after clustering, and the calculation results can reflect the dependence of municipal governments on specific policy tools. The topic prevalence is calculated with Formula (1):

(1) $$P_{k}=\frac{\sum_{i}^{N}\theta_{ki}}{N}$$

In Formula (1), *P_k_* represents the prevalence of topic *k*, *N* is the total number of policy texts, and *θ_ki_* refers to the probability of the topic k in the overall policy texts.

Second, we examined the balance of policy mixes across cities. In this way, we identified different configurations that represented the building blocks of policy tools designed to deal with the pandemic. We examined the balance of policy mixes by assessing the distributions of the policy tools included in each mix [22,26].

Third, we employed an ordinary least squares (OLS) regression model (Formula (2)) to investigate the relationship between a policy mix and a city’s pandemic response outcomes. The dependent variables were measures of the immediate outcomes of a city’s policy strategy: the number of major outbreaks in a city, the number of high-risk areas in a city, the number of secondary accidents in a city, and the city’s 2-year average economic growth rate. Given the fact that except the case of Wuhan, the mortality from the COVID-19 is insignificant in Chinese cities (for example, from 17 May 2020 to 1 April 2022, only 4 death cases were reported in total by 3 different cities; see www.nhc.gov.cn, accessed on 21 March 2022), which does not constitute a major concern in public opinion. In contrast with international practice, in the four dimensions we considered in this study, outbreaks, high-risk areas, secondary accidents, and economic consequences, we did not include mortality in the dependent variables. The independent variables were the policy tools used to deal with the pandemic, which were measured by the prevalence of each tool in the policy mix of that city. The city’s GDP, permanent population, number of universities, and digital city ranking were the control variables:

(2) $$dependent_{i}=\alpha +\sum \beta_{i}independent_{i}+\sum \gamma_{i}control_{i}+\varepsilon_{i}$$

## 3. Results

### 3.1. Identifying Policy Tools

Applying topic modeling, we obtained 28 topics that outlined the basic parameters of the municipal-level COVID-19 pandemic responses. In decreasing order of prevalence, these 28 policy tools are shown in Table 1.

The above 28 topics were divided into 3 categories based on the policy goals they were intended to accomplish:

1. Restrictions: These are policy tools that impose obligations, limitations, and prohibitions on individuals and collective actors [27]. These coercive measures aim to control the spread of the virus by reducing contacts and interactions. However, they can have negative effects [2] such as seriously impacting economic activity, supply chains, and public access to normal medical treatment. The restriction-based policy tools are as follows: lockdowns, quarantines, school closures, workplace and retail shop closures, canceling public events, gathering restrictions, travel restrictions, commercial activity restrictions, social distancing, mask requirements, and restrictions on government services.
2. Health protection measures: These are policies aiming to protect people from the direct effects of COVID-19. These proactive measures can alter and reduce the magnitude of a pandemic [27]. Compared with restrictions, which are obligations imposed on individuals and enterprises, health protection measures are responsive policy investments by the government. This category includes the following tools: information management, public testing, contact tracing, emergency investment in health care, sanitizer policies, vaccination services, monitoring population health, social mobilization, and improving the local risk response systems.
3. Socioeconomic support measures: These are policies that aim to protect the affected populations from the negative socioeconomic impacts of the pandemic and the secondary effects caused by restrictions [2]. This category consists of living/income support, debt or contract relief for households and enterprises, funding or fiscal stimulus, other work and production resumption support besides economic support, supply chain management, support for public access to normal medical treatment, other humanitarian assistance besides access to medical facilities, and the provision of e-government services.

No city relied exclusively on only one or two policy tools to deal with the pandemic; most deployed all of the 28 tools over the last 2 years with different distributions of these measures. Eleven cities (including Shanghai, Guangzhou, Hefei, and Baise) have tried to guarantee a minimum level of government services by not closing some service locations, such as petition reception and administrative approval services. Two cities (Chongqing and Xi’an) did not provide any public access to normal medical treatment facilities. Xi’an encountered a severe secondary medical disaster during its major outbreak in January 2022. Several uninfected people died owing to a lack of timely access to medical treatment.

### 3.2. Policy Mixes’ Tool Type Balance

The balance of a policy mix is measured by the distribution of policy tools within the mix. At the global level, the balance of the policy mixes is illustrated by the above sequential list. From this list, we identified three general trends.

First, the municipal governments opted for a preventive approach [28] to deal with the COVID-19 pandemic. This was indicated by the five most intensively used policy tools: information management, monitoring population health, mask requirements, sanitizer policies, and public testing. Second, social mobilization was not a prevalent policy tool; it ranked 18th among the 28 policies. This finding agrees with the low level of public participation in fighting the COVID-19 pandemic in China. Chinese crisis management has long been characterized by a government-led style, which heavily depends on state responses and investments [11]. The important role of social capital and resources has been minimized in the current Chinese crisis response policy design [29,30]. Third, lockdowns (ranking 24th) were not an important policy tool for cities, nor were economic supports (ranking 12th, 19th, and 26th) or supporting normal medical access for uninfected people (ranking 25th). 

**Figure 2:**
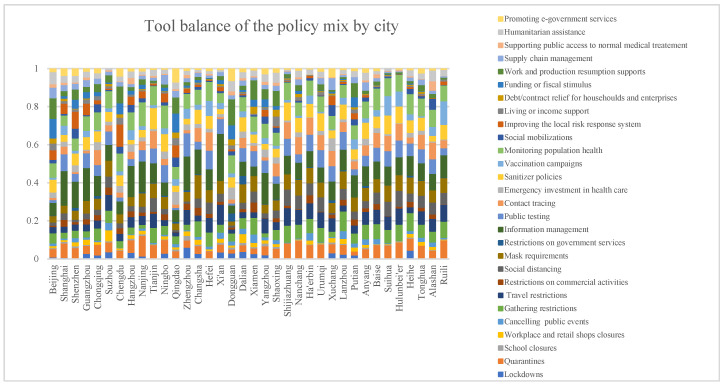
Tool balance of the policy mixes by city.

Although there were similarities, the distribution of these policy tools varied widely among the cities. The balance of policy mixes for the 36 cities is shown in Figure 2. The value of each color bar represents the proportion of that policy tool in the policy mix of the city. The proportion is measured by the prevalence of each policy tool in the policy mix of that city (for the results data, please see Table S1).

**Figure 3:**
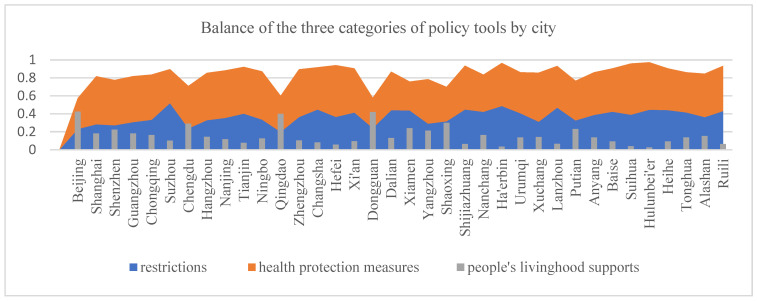
Balance of the three categories of policy tools by city.

To further explore how the cities incorporated different policy tools to deal with the pandemic, we analyzed distinguishing combinations of the three policy tool categories discussed above. The proportion of policy tools (the sum of the proportion of each tool belonging to the same category) in each group (i.e., restrictions, health protection measures, and socioeconomic supports) was calculated for each city (Figure 3). Based the result obtained, the integration of the three policy tool clusters could be categorized into five patterns representing the different policy choice strategies.

*Integrated health protection and socioeconomic support*. In this strategy, municipal governments attached more importance to people’s health and livelihoods. The policy responses centered on measures to protect people from the socioeconomic impacts of the pandemic. There was less emphasis on restrictions. Beijing, Chengdu, Dongguan, and Qingdao showed this pattern.

*Integrated restriction and health protection measures*. This strategy combined intensive restrictions with high-level health protection policy investments to protect the population from contracting COVID-19. Socioeconomic supports were less important in this policy mix. The following 15 cities showed this pattern: Changsha, Xi’an, Dalian, Shijiazhuang, Nanchang, Har’erbin, Urumqi, Lanzhou, Anyang, Baise, Hulunbei’er, Heihe, Tonghua, Alasha, and Ruili.

*Restriction-oriented response*. This policy strategy centered on restrictions and coercive measures to limit individual and business activities. Public health protection was less important, and socioeconomic supports were little utilized. Suzhou and Xiamen showed this pattern.

*Health protection-oriented response*. This policy mix emphasized investments in health protection measures that would protect people from contracting COVID-19. This strategy placed less emphasis on restrictions. It only slightly utilized socioeconomic supports, such as alleviating economic difficulties and the demands of daily life (such as procuring food supplies and access to normal medical treatment) during the outbreak. Shanghai, Shenzhen, Guangzhou, Chongqing, Hangzhou, Nanjing, Tianjin, Ningbo, Zhengzhou, Hefei, Yangzhou, Xuchang, and Suihua showed this pattern.

*Comprehensive policy mix.* This strategy used a balanced distribution of restrictions, health protection measures, and socioeconomic support policies. Shaoxing and Putian showed this pattern.

Maggetti and Trein (2022) found that politico-administrative arrangements did not markedly influence governments’ policy choices when dealing with the COVID-19 pandemic. Similarly, we did not observe any significant correlations between a city’s characteristics (i.e., economic, cultural, demographic, and geographic) and its policy design choices. For example, similar cities, such as Suzhou and Hangzhou, adopted very different policy strategies, while different cities, such as Beijing and Dongguan, adopted similar policy strategies. The policy decisions in many cases might simply be related to the local leaders’ personal preferences.

### 3.3. Effects of Policy Mix on a City’s Pandemic Response Outcomes

The R^2^, F, and *p* values obtained from the OLS model (Formula (1)) indicated that the model passed the stability test and is highly representative. The results can be summarized as follows (Table 2):

1. Among the policy tools, lockdowns, school closures, canceling public events, travel restrictions, social distancing, contact tracing, vaccination campaigns, improving the local risk-response systems, and supply chain management had significant negative impacts on cities’ 2-year average economic growth rates. By contrast, mask requirements, public testing, emergency investment in health care, debt/contract relief for households and enterprises, and support for public access to normal medical treatment did not have negative effects on the 2-year average economic growth rate.
2. Restriction-based tools did not significantly influence the number of major outbreaks or of high-risk areas.
3. Emergency investment in health care, vaccination campaigns, improving the local risk-response systems, supply chain management, and public access to normal medical treatment were negatively correlated with the numbers of major outbreaks, high-risk areas, and secondary accidents. Additionally, except for supply chain management, the others were positively correlated with the 2-year average economic growth rate.
4. By comparing these three policy tool categories, we found that restrictions and socioeconomic support measures did not significantly influence the control of the pandemic. In contrast, health protection measures strongly contributed to controlling the spread of the virus. Socioeconomic support measures particularly contributed to reducing the occurrence of secondary accidents.

## 4. Discussion

This study investigated how policy mixes affected cities’ pandemic management outcomes under the context that pandemic prevention and control measures have become normalized in China. The situations of the 36 studied cities differ from that of Wuhan, which was the first city to face a COVID-19 outbreak. The included cities were more prepared and were able to rely on information and practical experiences from previously affected cities. There were relatively fewer uncertainties and cognitive blind spots about the COVID-19 virus characteristics. Therefore, the effects and effectiveness of different policy mixes were comparable among the studied cities.

### 4.1. Relation between Policy Mix and Pandemic Response Outcomes

The logistic regression analysis results showed that restriction policies had no significant influence on controlling the pandemic. However, health protection measures were associated with controlling the spread of the virus. The results revealed a negative correlation between restriction measures and the economic growth rate. Restrictions and socioeconomic support policies had opposite effects on the number of secondary disasters. The more frequently and stringently restriction policy tools were used, the more secondary accidents and disasters occurred. Conversely, more frequent and intense use of socioeconomic supports led to fewer secondary disasters.

Based on these findings, we established a correlative relationship between a city’s policy mix pattern and its pandemic response outcomes (Table 3). Strategies 1 and 5 are the preferred policy choices for managing the pandemic. They are effective strategies for preventing or controlling the virus spread while avoiding the occurrence of serious negative socioeconomic consequences. Strategy 4 is suboptimal because although it is effective in controlling the spread of the virus, its consequences for secondary disaster prevention and economic protection are uncertain. Strategy 2 can control viral spread, but it might generate high levels of secondary disasters and have negative economic consequences. Strategy 3 is the worst choice because the restriction-oriented strategy cannot effectively control viral spread, but it negatively impacts secondary disasters and economic development.

### 4.2. The Importance of a Timely Lockdown

COVID-19 is highly contagious and can be spread through asymptomatic transmission; therefore, it is very difficult to prevent outbreaks. Many Chinese cities have been hit by the COVID-19 pandemic over the past 2 years. However, these cities have differed in the scale of outbreaks. Some cities have had a large number of cases across widely affected areas and have taken a long time to control the outbreak. Other cities have succeeded in controlling outbreaks in a short time with fewer cases and smaller affected areas. The implementation of timely lockdowns might be the key explanatory factor for these differences. A comparison between Shenzhen and Shanghai helps illustrate this hypothesis. The two cities are both international metropolia. The economic ranking of Shenzhen was the third (with 2.77 trillion yuan), and Shanghai is classified as the first (with 3.87 trillion yuan), among 337 Chinese municipal cities in 2020; Shenzhen is the fifth most populous city (with18 million permanent population in 2020) in China, and Shanghai is the second most populous city (with 25 million permanent population in 2020). Neither Shenzhen nor Shanghai had been hit by the COVID-19 outbreak until the arrival of the omicron virus in January 2022. Omicron is less dangerous but much more contagious than the previous variants such as alfa and delta.

Contrary to popular belief, not all Chinese cities implemented severe restrictions such as lockdowns to control viral spread. This is evidenced by lockdown policies ranking 24th among the 28 most often used policy tools (Figure 1), indicating that many municipal governments were reluctant to impose these measures. Some cities, including Shenzhen and Shanghai, refrained from adopting stringent restrictions to avoid their negative economic consequences. During the most recent outbreaks in the two cities in March 2022, in the early phase, both cities hesitated to impose lockdown measures for economic reasons. When the number of cases significantly increased for three consecutive days, Shenzhen imposed a lockdown policy, and the outbreak was under control one week later. In early March, the outbreak situation in Shanghai was similar to that in Shenzhen, but the authorities in Shanghai delayed the implementation of lockdown measures; they announced a lockdown policy 3 weeks after Shenzhen. As a result, Shanghai missed the optimal timing for initiating a lockdown. At present, the outbreak in Shanghai remains out of control (Table 4).

An appropriate and timely lockdown might be more effective than other restriction policies for controlling the spread of an outbreak, especially for severe viruses with a relatively high R0 (basic reproduction number) [2,31]. It might also be the least costly strategy, because when an outbreak is out of control, more cases and secondary disasters take place, resulting in more deaths and increased socioeconomic damage.

### 4.3. A Comprehensive Policy Mix for Compound Crises

The COVID-19 pandemic is a compound disaster in nature. It threatens not only public health but also multiple life-sustaining systems, functions, and infrastructure [32], such as economic activity, the supply of everyday necessities, the function of medical facilities, and logistics and transport systems. A pandemic is not a single negative event; rather, it is a concatenation of related negative events, generating multiple effects that become apparent on various time scales [32,33]. Pandemics are often accompanied by a variety of secondary disasters such as economic crisis, increased poverty, shortages of necessities, and excess mortality.

Studies have partly revealed the immediate negative effects that have arisen during the COVID-19 pandemic from medical, psychological, and social perspectives [34,35,36,37]. For instance, according to a recent study, during January–March 2020, in Wuhan, mortality from chronic noncommunicable diseases increased by 21%; there was an 83% increase in deaths from diabetes and a 66% increase in suicides [36]. The current study also found high incidence rates of secondary disasters during the pandemic period. In addition, larger outbreaks resulted in a greater number of secondary accidents. During the outbreaks in Tonghua, Xi’an, and Shanghai, a shortage of everyday necessities and excess deaths caused by restriction policies or the lack of medical access constituted two types of prominent immediate secondary disasters caused by ineffective supply chain management and a rigid “one size fits all” approach to pandemic control.

The complexity of the pandemic requires decision-makers to take a comprehensive approach rather than a single-event management approach to deal with the COVID-19 crisis [32,38]. Pandemic prevention and control are certainly the core aims, but decision-makers should also seriously consider the prospect of secondary disasters and enact compensatory measures to prevent the potential negative consequences of pandemic control measures, particularly restrictions [2,39]. It is necessary to implement a comprehensive and coherent policy mix with an appropriate distribution of the different types of policy tools available [40,41].

### 4.4. Study Limitations

This study has some limitations that should be addressed in future research. First, we examined only some of the serious immediate outcomes brought about by the different policy design choices. These outcomes were the most resounding concerns in Chinese public opinion. As such, we did not examine other issues such as the direct and indirect mortality caused by the COVID-19, which is an important concern at the international level. More comprehensive studies are necessary to provide a more in-depth assessment of the policy response outcomes and identify the optimal policy mix, as well as latent policy blind spots and mistakes. Such an in-depth study would better inform decision-makers and improve their policy responses when dealing with severe pandemics such as the COVID-19 crisis.

Second, given the complexity of our study (we investigated the effectiveness of the different combinations of 28 policy tools adopted by 36 cities), and for technical feasibility, we did not examine how the sequence and stringency of different policy tools affects the pandemic response outcomes. This limitation might be complemented by small-N cases with a limited number of policy tools or by detailed case studies.

Third, the completeness of the collected data regarding municipal policies was largely influenced by the amount of information that was made publicly available by the local governments. We were unable to discern precisely how many and what types of policies have been implemented in the form of internal governmental documents without informing the public. Moreover, since there was no central registry from which all data were collected using the same methodology, potential bias might have been introduced into the research. Although the results of our analysis are in agreement with our empirical observations, we recommend that future studies use integrated research methods [42,43] to overcome the limitations of the quantitative approach when investigating complex issues such as pandemic management.

## 5. Conclusions

This study investigated the relationship between the policy design of a city and its pandemic response outcomes. It contributes to the literature on comparative policy responses to a pandemic by offering a novel, local-level perspective.

Our analysis revealed that restriction measures did not significantly influence the spread of the virus, but they were negatively correlated with a city’s economic growth rate. In contrast, health protection measures strongly contributed to controlling viral spread. Intensive socioeconomic support measures, such as improving supply chain management and public access to normal medical treatment, reduced the occurrence of secondary disasters. The most effective policy strategy to deal with the COVID-19 pandemic appears to be a comprehensive policy design with a mix of restrictions, health protection measures, and socioeconomic support policies accompanied by a timely lockdown.

Our empirical findings can help to improve policy design by highlighting diverging patterns of policy strategies and their consequences, thereby generating useful lessons for how local governments should deal with similar crises in the future. We suggest that decision-makers take a comprehensive approach rather than a single-event management approach to deal with compound crises such as the COVID-19 pandemic. They should seriously consider the prospect of secondary disasters and enact compensatory measures to prevent the potential negative consequences caused by crisis control measures. Given the specific cultural and political characteristics of the investigated cities, these findings might not be applicable to cities beyond mainland China. We suggest further in-depth studies to investigate the relationships between policy strategies and their consequences by considering the specific local or national sociocultural and political contexts.

## Figures and Tables

**Table 1:** Key policy tools adopted by the cities.

Policy Tool	Illustrative Action	Prevalence
1. Information management	Providing public information; requiring residents to report to their communities if they had been to risk areas	0.104059
2. Monitoring population health	Measuring temperatures; checking health QR codes and travel codes	0.085552
3. Mask requirements	Mask requirements in public places and collective locales such as workplaces, buses, subways, and taxis	0.069997
4. Sanitizer policies	Disinfections in public places and collective locales; frequent hand-washing campaigns	0.069543
5. Public testing	Mandated nucleic acid testing for urban residents every one to three days	0.067159
6. Quarantines	Mandated isolation for close and indirect contacts; isolation or home quarantine for travelers	0.066421
7. Gathering restrictions	Restrict gatherings to a maximum of five people; ban all social gatherings	0.064547
8. Travel restrictions	Inner city traffic restrictions; cancel interregional travels	0.064093
9. Contact tracing	Epidemiological survey professionals question individuals and analyze travel information using big data methods to determine virus spread paths and identify close and indirect contacts	0.061482
10. Social distancing	Keep one meter distance in public or collective places	0.052455
11. Vaccination campaigns	Set up free vaccination sites in individual communities; require local cadres, social workers, and medical professionals to visit households to mobilize people for COVID-19 vaccine uptake	0.040704
12. Work and production resumption supports other than economic support	Facilitate permit approval for logistics vehicles; simplify administrative examination and approval procedures; provide employment recruitment services for enterprises	0.029407
13. Humanitarian assistance other than access to medical facilities	Open psychological comfort hotlines; local cadres and social workers visit vulnerable groups such as elders, disabled, and migrants	0.026284
14. Supply chain management	Enacting material supply plans; ensuring smooth transportation of medical materials and life supplies	0.021913
15. Emergency investment in health care	Booster medical supplies; purchase protective equipment for health staff; support manufacturing of testing equipment	0.021459
16. Improving the local risk response system	Require local governments or enterprises to improve emergency plans; enhance emergency drills and local risk screening	0.018904
17. Restrictions on commercial activities	Limiting customer or visitor flow in shopping malls, supermarkets, cinemas, parks, and tourist spots	0.01669
18. Social mobilization	Appeal to the voluntary participation of individuals and businesses to help fight the pandemic	0.015782
19. Funding or fiscal stimulus	Release funds to alleviate the economic impact of COVID-19; tax reduction and exemption	0.015441
20. Cancelling public events	Postpone sporting competitions; cancel expositions and festivities	0.01442
21. Promoting e-government services	Provide online government services such as social insurance and online administrative examination and approval	0.013057
22. Workplace and retail shops closures	Close retail outlets until further notice; permit only delivery and take-out at restaurants	0.012433
23. Living or income support	Distribute daily necessities to elders, disabled persons, and migrants; distribute consumption coupons	0.011467
24. Lockdowns	Full-scale lockdowns; district (partial) lockdowns	0.009878
25. Supporting public access to normal medical treatment;	Open green channels for dialysis patients, cancer patients, and pregnant women for medical treatment; inform doctors to prescribe adequate medication for particular patients during the period of lockdown	0.009424
26. Debt/contract relief for households and enterprises	Postpone households’ and enterprises’ debt and rent payments for three months; rent exemption in the period of lockdown	0.007777
27. School closures	Close schools and universities in the period of lockdown	0.005166
28. Restrictions on government services	Close petition reception; close marriage registration services	0.004485

**Table 2:** The effects of policy mix on the pandemic response outcomes.

		N-Outbreak	N-High Risk_Areas	N-SEC. Accidents	ECO_RATE
Restrictions	Lockdowns	0.152	1.975	1.010	−0.006 **
		(0.195)	(1.551)	(0.807)	(0.002)
	Quarantines	−0.036	0.038	−0.095	−0.000
		(0.045)	(0.360)	(0.188)	(0.000)
	School closures	−0.391	−4.433	0.560	−0.032 **
		(1.071)	(8.530)	(4.439)	(0.009)
	Workplace and retail shops closures	−0.231	−0.606	−0.806	0.001
		(0.158)	(1.258)	(0.655)	(0.001)
	Cancelling public events	0.042	0.912	1.171	−0.006 **
		(0.211)	(1.682)	(0.875)	(0.002)
	Gathering restrictions	−0.039	−0.676	−0.151	−0.001
		(0.094)	(0.748)	(0.389)	(0.001)
	Travel restrictions	0.100	0.412	0.039	−0.004 **
		(0.131)	(1.047)	(0.545)	(0.001)
	Restriction on commercial activities	0.011	−0.125	−0.533	0.003
		(0.168)	(1.339)	(0.697)	(0.001)
	Social distancing	−0.042	−0.551	0.753	−0.009 **
		(0.324)	(2.578)	(1.342)	(0.003)
	Mask requirements	0.110	−0.086	−0.611	0.009 **
		(0.263)	(2.096)	(1.091)	(0.002)
	Restrictions on government services	0.323	−0.171	0.547	0.003
		(0.188)	(1.494)	(0.778)	(0.002)
Health protection Measures	Information management	−0.024	0.026	0.057	0.000
		(0.028)	(0.226)	(0.118)	(0.000)
	Public testing	0.011	−0.204	−0.214	0.003 **
		(0.104)	(0.826)	(0.430)	(0.001)
	Contact tracing	−0.117	0.133	0.546	−0.007 **
		(0.238)	(1.895)	(0.986)	(0.002)
	Emergency investment in health care	−0.184	−0.434 ***	−0.373 **	0.005 **
		(0.129)	(1.031)	(0.537)	(0.001)
	Sanitizer policies	−0.000	0.815	−0.252	0.002
		(0.151)	(1.200)	(0.625)	(0.001)
	Vaccination campaigns	−0.016	−0.230 **	−0.062 ***	−0.012 **
		(0.032)	(0.256)	(0.133)	(0.000)
	Monitoring population health	0.068	−0.046	−0.053	0.000
		(0.052)	(0.411)	(0.214)	(0.000)
	Social mobilizations	0.119	0.826	0.211	0.002
		(0.188)	(1.497)	(0.779)	(0.002)
	Improving the local risk response system	−0.080	−0.558 **	−0.293 **	−0.003 *
		(0.164)	(1.307)	(0.680)	(0.001)
Socioeconomic supports	Living or income support	0.022	−0.911	0.378	−0.003
		(0.161)	(1.282)	(0.667)	(0.001)
	Debt/contract relief for households and enterprises	0.171	0.853	−0.715	0.014 **
		(0.449)	(3.577)	(1.862)	(0.004)
	Funding or fiscal stimulus	−0.022	−0.547	−0.209	−0.001
		(0.099)	(0.785)	(0.409)	(0.001)
	Work and production resumption supports other than economic support	−0.005	0.848	0.201	0.000
		(0.101)	(0.808)	(0.420)	(0.001)
	Supply chain management	−0.003	−0.236	−0.032 **	−0.009 **
		(0.282)	(2.243)	(1.167)	(0.002)
	Supporting public access to normal medical treatment	−0.046	−0.052 ***	−1.108 **	0.010 **
		(0.297)	(2.369)	(1.233)	(0.002)
	Humanitarian assistance other than access to medical facilities	0.000	−0.169	−0.396	0.000
		(0.063)	(0.503)	(0.262)	(0.001)
	Promoting e-government services	0.142	1.002	−0.222	0.000
		(0.115)	(0.918)	(0.478)	(0.001)
Control Variables	GDP	0.000	0.000	0.000	0.000
		(0.000)	(0.001)	(0.000)	(0.000)
	Permanent population	−0.001	−0.005	−0.005	0.000
		(0.001)	(0.009)	(0.005)	(0.000)
	University	0.015	0.154	0.098	0.000
		(0.035)	(0.279)	(0.145)	(0.000)
	Digital_city	0.017	0.069	−0.094	0.001 ***
		(0.027)	(0.214)	(0.111)	(0.000)
	_cons	−1.385	−10.244	5.165	−0.014
		(2.613)	(20.818)	(10.835)	(0.021)
*p*		0.346	0.878	0.716	0.102
r2		0.951	0.836	0.890	0.982
F		1.830	0.479	0.757	5.071

Note: *, **, and *** represent the 10%, 5%, and 1% significance levels, respectively. N-outbreak: The total number of major outbreaks; N-high risk areas: The total number of high-risk areas; N-Sec.accidents: the total number of secondary accidents; Eco-rate: 2-year average economic growth rate.

**Table 3:** The relationships between policy mixes and pandemic response outcomes.

	Policy Outcomes	Virus Spread Controlling	Secondary Disasters	Economic Consequences
Policy Strategy				
1. Integrated health protection and people’s livelihood support		Positive	Negative	Negative
2. Integrated restrictions and health protection measures		Positive	Positive	Positive
3. Restriction-oriented policy response		No correlation	Positive	Positive
4. Health protection-oriented strategy		Positive	No correlation	No correlation
5. Comprehensive policy mix		Positive	Negative	Negative

**Table 4:** Case comparison between Shenzhen and Shanghai.

	Date	12 March	13 March	14 March	15 March	16 March	20 March	21 March	28 March	1 April	26 April
Cases											
Shenzhen		66	86	60	92	91	44	28	9	2	0
Shanghai		65	169	139	202	158	758	896	4477	24,943	16,980
Lockdown				Shenzhen, start			Shenzhen, end			Shanghai, start	

Note: date was retrieved from the two cities’ CDC website (www.shenzhencdc.cn; www.scdc.sh.cn, accessed on 21 March 2022).

## Data Availability

The data presented in this study are available on request from the corresponding author.

## Supplementary Materials

The following supporting information can be downloaded at: https://www.mdpi.com/article/10.3390/ijerph19138094/s1, Table S1: The distribution of policy tools by city.
